# Managing the COVID-19 Pandemic: Experience of Managers in Healthcare: A Narrative Inquiry

**DOI:** 10.3390/healthcare12040447

**Published:** 2024-02-09

**Authors:** Angela Tolotti, Sarah Jayne Liptrott, Loris Bonetti, Shaila Cavatorti, Davide Sari, Luigi Caoduro, Annette Biegger, Alessandro Bressan, Dario Valcarenghi

**Affiliations:** 1 Nursing Development and Research Unit, Oncology Institute of Southern Switzerland, Ente Ospedaliero Cantonale (EOC), via Gallino 12, 6500 Bellinzona, Switzerland; angela.tolotti@eoc.ch (A.T.); loris.bonetti@eoc.ch (L.B.); dario.valcarenghi@libero.it (D.V.); 2 Department of Nursing, Regional Hospital of Bellinzona e Valli, Ente Ospedaliero Cantonale (EOC), via Gallino 12, 6500 Bellinzona, Switzerland; shaila.cavatorti@eoc.ch; 3 Department of Nursing, Oncology Institute of Southern Switzerland, Ente Ospedaliero Cantonale (EOC), via Gallino 12, 6500 Bellinzona, Switzerland; davide.sari@eoc.ch; 4 Department of Nursing, Pediatric Institute of Southern Switzerland, Ente Ospedaliero Cantonale (EOC), via Gallino 12, 6500 Bellinzona, Switzerland; luigi.caoduro@eoc.ch; 5 Nursing Direction Department, Ente Ospedaliero Cantonale (EOC), viale Officina 3, 6500 Bellinzona, Switzerland; annette.biegger@eoc.ch; 6 Hospital Directorate, Regional Hospital of Bellinzona e Valli, Ente Ospedaliero Cantonale (EOC), via Gallino 12, 6500 Bellinzona, Switzerland; alessandro.bressan@eoc.ch

**Keywords:** narrative inquiry, COVID-19, nurse administrators, narration, organization, administration

## Abstract

The pandemic represented a challenge for hospital managers at different levels, required to reorganise services without compromising care. This study aimed to analyse the experiences of hospital managers during the COVID-19 pandemic. A narrative inquiry was conducted in a multisite acute hospital. Data were collected through narratives and open-ended questions. Direct and non-direct-care managers were invited to participate. Data were analyzed considering Clandinin & Connelly’s (2000) framework and Braun & Clarke, (2006). Thirty-six narratives and open-ended question responses were analysed. Participants were nurses (*n* = 20), doctors, technicians, administrative and hospitality service managers. Themes were grouped into three narrative dimensions: (1) personal—“the emergency engulfed us”, (2) practical/professional—“managing the pandemic”, (3) social—“the strength of the team and people”. Different narrative threads were identified between direct-care and non direct-care managers. Problems faced, factors helpful for management and suggestions for improvement were also reported. The pandemic had an important impact on managers and their roles, in terms of the need for clear concise information, staff support, and adequate professional and technical resources. A sense of unity and belonging facilitated management.

## 1. Introduction

Described as a threat to global health [1], the Coronavirus Disease 2019 (COVID-19) first reported in December 2019 [2], now reports 505,035,185 cases worldwide with 6,210,719 deaths as of 21 April 2022 [3]. Globally, healthcare systems have responded to an unparalleled call to address the healthcare needs of vast numbers of individuals affected by COVID-19, requiring reorganisation of services, and re-allocation of resources [4,5,6].

Staff working in healthcare settings have described physical and psychological effects of the pandemic, often reporting concerns and anxiety over inadequate or lacking Personal Protective Equipment (PPE), and contracting or transmitting the infection to patients or family [7,8,9,10]. Uncertainty was increased by the evolving situation, lack of timely communication and continued changes to practice [11]. Social isolation measures meant that inpatient care further intensified to incorporate additional emotional support replacing that previously provided by families and carers [12]. Moreover, staff redeployment, for some into intensive care specialities, left healthcare workers reporting stress, anxiety and feelings of inadequacy in care delivery [7,13].

Amid times of emotional burden, individuals rely on leadership for guidance [14] and healthcare managers have a pivotal role [15], supporting staff whilst ensuring services are not compromised. Leadership may affect psychological distress in employees [16] and its absence may lead to breakdown of trust and psychological safety [17]. Successful leadership through a crisis depends on open communication and collaboration [18], however challenges arise where managers themselves are confounded by uncertainty. As well as being personally affected by the pandemic, managers are in a unique position having to respond to both staff and organisational needs. Literature investigating the impact of COVID-19 on working practices across all occupations indicates a range of managerial challenges including provision of support systems for workers, managing resource limitations and supply chain management, remote working, respecting physical distancing requirements and human resource management [19,20]. Within the healthcare setting, challenges translate into a responsibility for implementing responsive care delivery models incorporating workforce planning strategies, and addressing issues relating to scope of practice and competency of staff [21]. Ensuring staff safety, psycho-emotional wellbeing, managing conflict and sustaining morale are described by nurse managers during the pandemic, with personal reflections on the role in terms of feeling unprepared, and overwhelmed by new tasks and duties [12,22,23]. It is evident therefore that persons in managerial roles of various levels also require support to lead in crisis situations, however support should be tailored to meet the requirements of the individual, and reflect peculiarities of context.

Hospital services in the canton Ticino in Switzerland are multi-centred across the region and centrally co-ordinated. In response to the COVID-19 pandemic, a significant re-organisation of services was conducted including identification of hospitals dedicated to care of patients affected by COVID-19 and mass redeployment of staff involving all hospital services. Managers were also faced with the unique challenge of managing a workforce heavily dependent on cross-border movement of hospital staff, dealing with border closures to limit virus spread and movement of workers [24]. In light of this situation, it was felt important to investigate the experiences and challenges faced by managers working at various levels within the organization.

The purpose of this study was:

- to understand the experience of managers during the pandemic
- to identify the difficulties and strategies used to face challenges within the managerial role during the pandemic
- to describe factors perceived as helpful for managers in facing this critical period and proposals how to better manage a crisis situation

## 2. Materials and Methods

A narrative inquiry research approach was chosen, according to Clandinin & Connelly [25]. The narration of the experience informs the construction of knowledge at personal, social and professional identity levels [26]. 

The study included participants from three public hospitals in Switzerland: a general hospital, an oncology hospital and a paediatric hospital. Potential participants were directors and middle managers—nurses, doctors, administrators, technicians and from the hospitality sector. The research team worked within the same three public hospitals.

Data was collected in the form of narrated stories, and responses to three guiding questions. A brief guide was provided on how to prepare the narrative (Supplementary File S1). Potential participants were invited to take part in the study through a web link, disseminated via institutional websites. Participants were informed of the aims of the study, voluntary participation, that their answers would be anonymised, and assured confidentiality. The online format was available from 30 April 2020 to 31 August 2020, when data saturation was achieved. Collected data was stored on a password protected server accessible only to the research team.

Data analysis of the narratives was performed according to the framework of Clandinin & Connelly [25]. Each narrative was read several times to gain an overview and understand it in its entirety. Each narrative was analysed by 3 experienced qualitative researchers, using the 3 levels of justification: personal, practical/professional and social, each mutually informing [27]. With regard to the first level of justification, threads of stories or narratives concerning the personal sphere such as thoughts, feelings, and emotions were identified. In the second level, the focus was on personal experience regarding the role played during the pandemic, highlighting what participants experienced and learned from a professional point of view. In the third level the narrative threads captured the experience of the people within the social dimension, the impact of their experience within the social context of the organisation and family, what was important and what had been learned. For each level, narrative threads were grouped into themes and parts of the narrative were identified, called micro-stories, which highlight the participants experiences during the pandemic. Narratives belonging to nurses and doctors (direct-care group), and the group of administrative, technician and hospitality service managers (non direct-care group) were analysed separately. Responses to guiding questions were analysed following a thematic analytical approach [28]. Analyses were performed by two experienced qualitative researchers.

The study involved healthcare professionals, and according to Swiss Human Research Act, Article 3 (2011, updated 2021) no formal ethics committee approval was required. Participants were asked to avoid the use of names within the text and reference specific circumstances that could identify the individual. To ensure confidentially and anonymity all identifying information was removed before processing the data.

## 3. Results

### 3.1. Participants

Two-hundred and fifty managers were invited to participate (179 doctors/nurses, and 71 administrative, technician and hospitality service). Thirty-six narratives were received and analysed (14.4% response rate): 20 from nurses, six medical staff, three technician, six administrative and one from a hospitality service (Table 1). 

### 3.2. Narratives 

The narratives were analysed separately (direct-care and non direct-care managers), and for each group, the main themes and narrative threads were identified for each level of justification (Table 2).

In both groups the same themes were identified but with similarities and differences in the narrative threads for each level of justification. We identified the following themes: “the emergency engulfed us” (personal dimension), “managing the pandemic” (practical/professional dimension) and “the strength of the team and the people” (social dimension). For each level of justification, micro-stories characterising the experience of the participants were selected. Each micro-story was entitled with the key words of the narrative (Table 3).

#### 3.2.1. Personal Level “The Emergency Has Engulfed Us”

Respondents narrated how the emergency had taken over everything—people, professionals, hospitals, citizens, families, feelings, security, and the lives of everyone. It was described as something that engulfs, and an invisible enemy to be fought.

In the narrative threads, both groups highlighted the difficulty of having a realistic perception of the problem, and of the seriousness of the pandemic, which initially appeared to be another countries problem. Instead images broadcast in the media played an important role in raising awareness of the problem—no longer being someone else’s problem. Direct-care and non direct-care managers described living in extreme uncertainty, experiencing fear and suffering. “Nothing was ever the same again” was recounted several times by direct-care managers, where life continued but also became stuck and everything was different. In both goups, narratives described how certainties collapsed and the fear of infection and contamination was predominant. Nurses also narrated how their lives had changed, in particular their professional life highlighting their personal sufferance when having to support patients in isolation, substituting family who could not be present. Both groups used war-like language, in particular nurses used the words “going to the front” to emphasise feelings. They declared themselves on the lookout, and ready to respond quickly to needs at work and home. The non direct-care managers described a “surreal” environment where change became a constant. 

#### 3.2.2. Practical/Professional Level: “Managing the Pandemic”

In both groups, narrative threads concerned the sudden change of routine, and difficulties linked to this new experience. Administrative managers narrated the need for continual change, with support from military services for the healthcare team and the possibility for nurses and doctors to stay in nearby hotels. Difficulties were described in reorganising the hospital structure and staff to cope with the emergency and the new needs that derived from it. Nurses stories highlighted the experience as unique and in a certain sense unimaginable, for which one was absolutely unprepared. Making choices was not easy, particularly when asking staff to change shifts or move units, often with little or no advanced notice. For the non direct-care managers, difficulties were described where they sometimes had to do things that they did not want to do.

In both groups, information played a fundamental role. For the direct-care group, managing one’s own team in the pandemic phase required taking on new aspects of the role such as gathering as much information as possible. This was important to allow the team to work safely and to have the situation under control, managing one’s own fears and at the same time reassuring the team. Often direct-care managers highlighted being submersed in information, sometimes contradictory and changing rapidly, making it difficult to select which information to share. In contrast non direct-care managers described difficulty in being able to respond to the questions of their teams due to the lack of information available. 

For the direct-care managers, the pandemic also represented a personal and professional challenge, where uncertainty and fear allowed people to learn from the experience. Some narrated how this led them to reflect on personal/professional values and the need to make lifestyle changes. Others pointed out how facing ‘dramatic’ situations was an opportunity to open up to something new, changing one’s way of thinking and experience what it means to share. These new situations with particular regard to patient care, were a stimulus to study and learn more. Both groups narrated the lack of PPE in the initial phase of the pandemic. One non direct-care manager described not only the difficulty of accessing PPE but personal suffering as he was unable then to provide this to staff who needed it.

#### 3.2.3. Social Level: The Strength of the Team and the People

Several elements affected groups at both intraprofessional and interprofessional levels such as the increase of knowledge and interprofessional collaboration. Another element was the consideration and understanding between staff within smaller groups, but also across the organization, showing a strong sense of unity and belonging. 

In both groups, narrative threads emphasized how awareness that the crisis situation involved everyone and at all levels, lead to a sense of unity and commitment that some nurses described as moving at times. Everyone moves in the same direction, towards common objectives, searching together for possible solutions to problems encountered. New forms of collaboration were being experimented with. 

Another socio-cultural issue reported by both groups was that of playing a leadership role. Reflections in this regard emphasize how the role of the leader was facilitated by collaboration and knowing that “one could count on the concrete support of so many people”. In a narration from the non direct-care managers, it was reported how being a leader in such a situation transmitted great energy.

Another element common in both groups was the importance of support from managers own managers, which was shown through concrete actions, proximity and support, where Senior Management attended hospital wards to be close to those on the front line. This created a strong bond between people with different roles and within different groups. In the direct-care group, the importance of support from family was also strongly narrated. 

Both groups highlighted a strong sense of organizational belonging. Nurses also emphasized how their professional values played a central role, giving everything to make it possible to deal with the dramatic situation they were experiencing in a ‘unique’ way. In the narratives an administrative manager focused on major lessons learned, the exceptional results achieved by the organization as a whole in dealing with this emergency. Having a common goal and a strong sense of urgency made it possible to overcome unitary visions and ‘entanglements’ that normal day-to-day routines hinder.

### 3.3. Responses to Guiding Questions 

In addition to completion of the narratives, respondents also answered one or more of the guiding questions (Supplementary File S1), relating to problems and strategies to deal with issues, factors helpful in facing the pandemic and proposals to better manage a pandemic situation. Summaries of findings are presented in Table 4, Table 5 and Table 6. 

Regarding problems and strategies to deal with issues (Table 4), three themes were identified: emotions, the managers’ role during the pandemic and communication flow & adapting to the situation. Many of the problems identified and strategies to deal with these problems were elaborated in the narratives, however some were novel aspects reported below. 

Regarding the theme of emotions, a sense of responsibility was described by both groups—for non direct-care managers this was being aware that everything depended on them, while for the direct-care managers there was greater awareness of routine responsibilities. Frustration was described in both groups of managers. For direct-care managers this was in terms of not being able to be with the staff caring for COVID-19 patients, the closed minded approach of some colleagues, and not having enough time to take the best decision. For the non direct-care group, this related to how priority was given to hospitals dealing with patients affected by COVID-19, while greater burden was left on those who remained to perform usual tasks. 

Regarding the managers role during the pandemic, both groups shared problems related to reorganisation. For direct-care managers this was shifting appointments, remote patient management, and revising treatment strategies, while for non direct-care managers this was closing outpatient activities. Both groups of managers described how their role included promoting personnel well-being be that through support, working flexibility and creating a positive climate. Nurses also highlighted the difficulty of performing dual roles—clinical and managerial, and having adequately trained staff for critical areas. One non direct-care manager described how despite personal feelings of tension, his role was to remain positive with his staff. 

Regarding factors reported to be helpful in facing the pandemic (Table 5), three themes were identified: personal characteristics, knowledge and competency, support from inside/outside of the organisation and ‘we’re all in the same boat’. In addition to the findings from the narratives, regarding the theme personal characteristics, both groups reported the importance of professionalism. They spoke of sharing and addressing issues together—for the direct-care managers this was with other managers in weekly meetings, while for non direct-care managers the creation of a chat group to share information was useful. In the theme ‘we’re all in the same boat’, other than that emerging from the narrations, both groups highlighted a positive working environment as a helpful factor. For nurse managers the welcome and support from Intensive Care Unit staff to redeployed nurses was particularly helpful during what was a professionally difficult situation.

Respondents were also asked to give suggestions based on their experience, of what could be improved in order to better manage a crisis situation (Table 6). Proposals common to both groups related to the provision of support—for those who experienced the virus at first hand and in terms of encouraging active communication, sharing emotions and experiences between managers and their teams. Another proposal was improving information and communication. While non direct-care mangers suggested better use of the hospital app to receive timely and updated documents and key information, direct-care managers suggested the use of summaries, filtering, and coordinating information provision to ensure it was concise, useful and pertinent. Having an emergency plan in place to deal with crises was important for both groups, with another joint proposal was greater awareness of technical resources, human resources and having adequately trained staff. Both groups felt there were lessons to be learned regarding patient care, with direct-care managers focusing on learning from what worked, sharing good practice, and developing transversal services, while non direct-care managers suggested better transmission of information regarding hospital safety to avoid missed care.

Direct-care managers also made suggestion such as avoiding bellic language, valuing both technical and relational competencies, avoiding the creation of false expectations in the general population, respecting the rules, and involving the right people in decision-making process. 

## 4. Discussion

The aim of our study was to understand the experiences of managers during the pandemic using narrative inquiry. Through narration, people can reflect on their experiences, drawing useful lessons from such that can be useful for each person but also for the organization as a whole. We also asked respondents to share their problems, factors facilitating their role and suggestions to improve management of a crisis. Results highlight the principal difficulties but also that which has allowed participants to face difficult situations as individuals and managers. 

Media and particularly social media, have been key instruments of communication during the pandemic, providing a significant quantity of news with variable quality of information [29,30]. This contributes to the challenge of having a realistic understanding of what is happening as described by both groups of participants in this study. The pandemic has impacted both at personal and social levels [31] with imposed changes, some of which remain and may not return to ‘normal’ within the foreseeable future [32]. Participants within this study agreed, describing their experience of a changed and unpredictable situation, never to be the same again. War metaphors were common within the narratives, in particular by nurses, having also been reported within the literature [33], and reflecting a sense of seriousness and urgency [34].

Within the main issues emerging, a key role noted at the ‘personal level’ was represented by emotions experienced, independently of the managers role. An emergency situation which was reported to have ‘engulfed’ respondents in this study, brought uncertainty, fear, and mirrors findings not only for managers but generally within the healthcare setting and beyond [22,35]. Fear of being a source of contamination for oneself, family or colleagues is commonly reported by staff dealing with patients affected by COVID-19 [8,9,19], however this was also observed across managers in this study, who may not have been in direct contact with patients. 

Nurses described personal suffering due to the emotional emptiness of patients, speaking of a surreal atmosphere in which they found themselves as substitutes, for the presence and comfort that is usually brought by family members, a finding also reported by managers in mental health centers [12]. Frustration of nurse managers was described, caused by being unable to be with their team, with similar reports in the literature where leaders described being powerless, able only to listen [36]. In response to the COVID-19 pandemic, staff were often redeployed and some centres had reserve teams to support high volume clinical activity and backfill staff in case of absence [37]. Without such support however, staff assume the burden and frustration of covering the remaining activities, as suggested by a non-direct care manager in this study.

The need to introduce organisational changes was described by both groups in this study. In particular, for nurse manager this referred to sudden and frequent changes, having to choose who to ask to change shift or to move units, as described elsewhere in the literature often with little or no advanced notice [22,38]. Similar issues with reorganisation of services were reported by direct and non-direct care managers, reflecting reports within the literature of how services were consolidated and prioritised in order to maintain operations with available resources [21]. 

Organisational changes often meant changes in roles, and it emerged that nurses were challenged by what had often become a dual role—not only managerial but also clinical, a finding reported by other nurse managers covering administrative and clinical roles as well [39]. A lack of staff experienced in the management of critical care patients is reported in the literature with, similarly to findings in this study, with attempts to support skill development by working with more experienced nurses [38], a welcoming environment was also described by nurse managers in the guiding questions to have been useful in this difficult situation. 

The speed with which information was transmitted, sometimes being contradictory, required adaptation to new situations, guidelines and procedures to follow. Scenario planning is virtually impossible [40] and it is therefore unsurprising that these managers described their difficulty. Confusion and uncertainty in this period is a common finding [11] as real-time dissemination of clear information to staff is an arduous task. Interestingly, this study highlighted how health professional managers described this period as information overload. Nurse managers in this study emphasised how such information challenges the impact this had in relation to doubts in their decision-making based on information available and the ethical and moral implications, contributing to existing literature on the subject [41]. Other non direct-care manager roles described a paucity of information limiting their ability to respond to questions posed by staff. Findings suggest modes of communication require revisiting to address each group’s needs. 

This unprecedented healthcare emergency has seen evidence of adaptation and resilience in healthcare staff [7,41], also described in this study by nurses, along with greater understanding between staff groups. Facing the emergency, and activating organisational and staff resources has been facilitated by the ability to share knowledge and skills internal and external to teams, and recognise and value creativity, despite this being perceived as unchartered territory for many. Fostering a supportive environment in which open communication and collaborative relationships are realised can promote effective decision-making [42,43], as suggested by both groups in this study. 

In both groups of managers, the sense of responsibility was evident, a finding that has been reported by nurse managers within the literature [44]. Non direct-care managers described the difficulty of maintaining staff morale, trying to show positivity and maintaining calm despite the tense situation as is described within the literature [14,22]. This sense of responsibility is also linked to the desire to ensure staff safety, which is a particular challenge in a pandemic. A focus on staff well-being was also observed, similar to findings in the literature including teamwork, communication, putting people first and helping with uncertainty [45,46]. Managers in both groups described their difficulty with timely access to PPE for their staff, and ensuring safety of those caring for patients affected by COVID-19 as reported elsewhere [7]. 

In terms of the ‘social level’, many of the themes here are resonant of those within disaster nursing literature including improvisation, collaboration, leadership, community support and crossing of boundaries in response to the crisis [47]. The unity of the team was reported by both groups of managers to be of importance, in line with the willingness of staff, colleagues and creation of reciprocal trust [18]. 

Being in the ‘same boat’ and moving in the ‘same direction’ highlighted the unity of groups working towards a common goal, as collaboration between professionals was key to resolving problems occurring [18]. There is evidence in the literature of how the COVID-19 has incited change in organisations, regarding new projects, new collaborations and overcoming barriers [48], and although this was also observed in this study, participants also reflected on why such barriers existed beforehand how the ‘common goal’ was the driver to surmount such difficulties. Peer support was described as beneficial in improving mental health during the COVID-19 pandemic [49] so it is not surprising that findings here from direct care managers described how sharing issues together was valuable.

Both groups of managers emphasised the decisive role played by the support and backing of the organization, of its managers and that took the form of being there and being available. While the managers role may be seen as one where difficult decisions are made that are not always shared by the team, nurse managers in this study described their experience of support from both their managers and their team. Perceived organisational support and supervisor accessibility are associated with job-related well-being and affective commitment to the organization [50], being areas to focus support in case of future emergencies. Support for healthcare workers from partners and family is also recognised [51] and was evident within in the group of direct care managers this group as a safe harbour allowing one to return to normality for a short while. 

Our findings showed the strong sense of belonging to the organization in both groups of managers, which was a winning factor in facing the difficult situation, giving motivation, strength, and the energy to face any obstacle or difficulty. It is suggested that sharing stressful experiences may promote cohesion between the employee and organization through emotional bonding and shared identity [52], surely a factor to build on in the future. 

In proposals to improve management of a crisis, both groups described the importance of having support—psychological support for team members caring for patients affected by COVID-19—as suggested in the literature [53,54], and the importance sharing emotions and the teams experiences [55]. Other proposals included effective communication as reported by Abi et al., [56], and having an emergency plan in place to deal with such situations, a theme mirrored within the literature [57]. Other shared suggestions included having adequate PPE learning from the experience and developing specific knowledge and competencies in order to be prepared for similar situations in the future, findings which are supported within the literature [7,58,59,60]. The process of reflection on the experiences of employees may contribute to organization learning and improve organizational know-how, impacting on employee behavior [61]. 

While this paper provides an in-depth view of the experiences of managers during the pandemic, it is recognised that there are limitations with the study. Despite an invitation to all managers to describe their experiences, just under 15% of invited participants responded. While this is a small number, a range of managers were represented providing their experiences. Participants were invited from just three hospitals across a multi-site setting. It is not possible to confirm whether the experiences of managers in hospitals closer to the Swiss-Italian border had similar experiences. One limitation of the study was the impossibility for researchers and participants to meet during the pandemic due to restrictions in relation to personal contact. 

## 5. Conclusions

Our study is focused on analysing the experience of managers involved in managing during the COVID-19 pandemic. The results revealed common aspects in experiences related to the initial difficulty in realistically perceiving the problem, which was underestimated, and the role of the media in fostering greater awareness. Fear of contamination, facing the unknown and the climate of uncertainty brought about important changes in both private and working lives. Changes also concerned language used, being war–like, where there is an enemy to defeat and a front on which to fight.

Managing during the pandemic meant having to make difficult choices quickly, relating to the reorganisation of services and activities, affecting staff who were required to be available and flexible. Information was a highly critical element and dealt with inadequately. Modes of communicating information should be adapted to respond to the needs of different managerial groups.

Challenges related to managers roles in such a complex context allowed them to learn and change established ways of thinking both at work and in their private lives. Organisational learning occurred both in the individual operational units and between the different organisational units. Dynamic environments were described in which intraprofessional and interprofessional collaboration took place. Working towards a single goal, each one providing knowledge and skills, allowed everyone to move in the same direction and strengthen the sense of belonging to the organisation, which was a key element and should be considered for the future. For all groups, the leader’s role in fostering a supportive working environment among peers and towards the team was fundamental. Leadership at various levels, that is sensitive to and able to promote these values, is an important factor in the development and success of a healthcare organisation. Future research may be directed towards the investigation of which styles of leadership may encourage these values in particular during crisis situations. 

## Figures and Tables

**Table 1:** Respondent demographics.

	*n* = 36 (%)
Gender	36
Male/Female	18 (50.0%)/18 (50.0%)
Working directly with patients affected by COVID-19	
Yes	11 (30.6%)
No	25 (69.4%)
Years in managerial role	
<5	13 (36.1%)
6–20	19 (52.8%)
21–35	1 (2.8%)
>36	3 (8.3%)
Living status	
lives alone	6 (16.7%)
lives with others	30 (83.3%)

**Table 2:** Level of justification—themes and narrative threads.

		Narrative Threads	
Level of Justification	Theme	Direct-Care Managers (*n* = 26) (Nurses/Doctors)	Non Direct-Care Managers (*n* = 10) (Administrative, Technical and Hospitality Service)
Personal	The emergency has engulfed us	it’s not always someone else’s problem negative emotions: fear, worries, suffering nothing ever the same again established routines are broken down certainties collapsed going to the front	it’s not always someone else’s problem negative emotions: fear, worries certainties collapsed news from the front surreal reality
Practical/professional	Managing the pandemic	sudden changes/new experience feeling unprepared, difficult choices information to keeping the situation under control information—too much changing ways of thinking to deal with the situation	sudden changes/new experience personnel and structural reorganisation information—too little doing what you didn’t want to do
Social	The strength of the team and people	we are all in the same boat leadership role support from others (staff, senior managers, family) sense of belonging	we are all in the same boat leadership role support from others (staff, senior managers) sense of belonging positive aspects of the emergency

**Table 3:** Micro stories.

Level of Justification Theme	Micro–Story Extracts
Personal The emergency has engulfed us	Micro-story n. 1 *“go to the front”* (extracts) *“But the most surprising thing was when I asked who would have wanted to go to the front…, as we call it. Yes the front,.. because for us it’s a war, war against an invisible enemy that not only kills but takes away feelings, kills memories…”* (Nurse Manager) *“…I remember very well the emotion of the last goodbye to the nursing team the day before they left for six weeks to work in the Covid hospital, we thanked them for their sacrifice, for their willingness, for the risk they were running… The news we received from the front was dramatic, with an unknown and very aggressive virus, with patients intubated for 3-4 weeks and in the worst cases they died without even being able to have their loved ones near them… I remember that it was said that this was an inhumane disease…”* (Administrative Manager) Micro-story 2 *“The war of the worlds”* (extracts) *“It’s collective hysteria”... he said without thinking much about it. I saw he was very busy and with lot’s of worries that took him far away from me.* *I was talking to him and I was actually interested in something else entirely. I needed to know what fate awaited my unit. So this problem captured all the attention of the person, but I would have liked him to have been interested in my problem instead.* *And well that’s how it was, this damn bug was taking over everything, like in the war of the worlds. Everything you touched, every person you met, it could lead you to disaster.* *And then I’d start to get out of breath and dizzy. And even though I wasn’t sick, I felt like it.* *Of course I’m rational and I’ve always been active and present at work, but in a corner of my mind there was always that voice saying, eh, no, this time we won’t get out of it…* *Instead out there, in the field, there were people who were really ill and people who really cared for them. And the knowledge of that made me find the oxygen”* (Medical Manager)
Practical/professional Managing the pandemic	Micro-story n. 3 *“Things you wouldn’t want to do”* (extracts) *“…given the impossibility to visit patients, family members would stand under the terraces of the hospital to see and communicate with their loved ones, it was moving, and my job was to have to tell them to leave…”* (Administrative Manager) Micro-story n. 4 *“Changing ways of thinking to face something new”* (extracts) *“Aware… I don’t want to forget the suffering that the pandemic created in patients, relatives, caregivers, and all of society in more or less intense ways.* *Personally, I found the lockdown period interesting. I find the experiences related to the crises a source of growth; the change of routine, having to face dramatic situations, changing our ways of thinking to face something new has left us to live and share moments never experienced before.* *During the most critical period I totally changed role, I left an outpatient department for the intensive care unit, an environment I’d left more than 20 years ago.* *The tension related to the new environment, the feeling of being insecure and not professionally ready for any situation led me to dedicate myself to study again.* *The welcome from the intensive care colleagues was fantastic. Their guidance during the first days and their constant support made sure that I never felt alone but always supported. The collaboration created was fundamental, so that in a short time I went from a state of tension to one of well-being, where the professional gaps became points of exchange (especially between colleagues on temporary loan) and to increase my professional knowledge.* (Nurse Manager)
Social The strength of the team and people	Micro-story n. 5 *“We are all in the same boat”* (extracts) *“during the COVID emergency this Spring, the positive aspect for me was the great collaboration that existed among healthcare professionals across the board, at times even moving given the very dramatic context…. the people with whom I work every day perceived, as I did, the difficult moment we had to overcome, and we immediately stood together to support each other and do everything possible… I repeat once again that the most important thing during this period was solidarity and standing together”* (Nurse Manager) Micro-story n. 6 *“Unity amongst all and at all levels”* *“our team responded magnificently to this enormous challenge. Having a common goal “managing this pandemic wave in the best way possible with as little damage as possible”, united not only our team but the whole organisation ”* (Administrative manager) Micro-story n. 7 *“The strength of the team”* (extracts) *“Leading a team and being even more of a leader in a crisis phase like we hadn’t experienced in decades, gave me and us a great buzz. The fact of having a great team really reassured me and made me confident that we would be able to handle this acute phase and that things would get better.”* (Administrative Manager) Micro-story n. 8 *“Everyone did their best”* *“I had a unique experience, for which no one was prepared. I was able to count on the contribution—support of my hierarchical and functional superiors first and foremost and my staff. I was able to benefit from the support of the other departments… I was able to see collaboration at all levels: from senior managers to hospitality services; from the technical service to IT. Everyone did their best to help us, the soldiers from Civil Protection and staff on loan at the tent. The dearest people were my loved ones “* (Nurse Manager) Micro-story n. 9 *“Desire to feel useful and part of it all”* (extracts) *“…I was lucky enough to have a team that compares to no other, everyone has given and is giving their best to cope with the thousands of requests and the daily evolution of the scenarios… each department head has shown a very high sense of responsibility towards their staff and the organization, but above all, not forgetting a strong attachment to the profession. We were able to move the outpatient unit in 2 days, bringing together two teams that had never worked together before, but with a spirit that distinguished nurses, commendable, all ready to do their part, all with the fantastic desire to feel useful and part of it all. The staff have responded with a willingness to be there, many have proposed to go to work in a Covid ward, so we have done our part, yes, because it was important to be there, to understand that the unity of nurses doesn’t only come from what they teach us in universities, but from that sense of belonging that is unique in the world, we are nurses.”* (Nurse Manager) Micro-story n. 10 *“Often the barriers are ourselves”* (extracts) *“…it’s interesting how in these occasions people’s creativity is productive and used to the maximum, decisions are quick, teamwork is optimal… it’s incredible what the (hospital name) was able to do in a short time… it’s normal to wonder why it can’t do the same in normal conditions… the explanation I gave myself is that in this phase we all had one common goal (to treat COVID patients with as little damage as possible)… the lives and the deaths of so many people were at stake… At that point it was easy to bring all stakeholders together under one roof… in ‘normal’ everyday life we often have lots of goals to achieve, some of which are divergent or even conflicting… the sense of urgency varies according to the leader and the situation, leadership is not always clear and direct, norms and habits often clog up the organization… I think that from a business and leadership point of view, COVID has taught us a lot. Also the fact that often the barriers are ourselves… “* (Administrative Manager)

**Table 4:** Problems and strategies to deal with issues.

Theme	Sub-Themes	Direct-Care Managers (*n* = 26) (Nurses/Doctors)	Non Direct-Care Managers (*n* = 10) (Administrative, Technical and Hospitality Service)
*Emotions*	Fear	becoming ill (P) infecting others (P)	becoming ill (P) not being able to transmit all the information to staff (P)
	Uncertainty in decision-making	doing the right thing (P) moral implications (P)	doing the right thing (P)
	Sense of responsibility	greater awareness of daily tasks (P)	everything depends on us (P)
	Frustration	sharing the suffering of patients in their loneliness due to isolation (P) not being able to be with the nurses caring for COVID-19 patients (P) the arrogance of some colleagues who look no further than their own reality (P) not having enough time to make the best decision (P)	priority given to other hospitals but greater burden left on those who remained to perform the same tasks (P)
*The managers role during the pandemic*	Clinical activity	shifting appointments (S) reorganisation of clinics (S) remote management of patients (S) modifying treatment strategies to reduce the risk for patients (S)	closure of clinics, inpatient units (P/S)
	Managing personnel	listening and supporting staff who feel unprotected (S) managing positively the fear of others (S) asking for collaboration and flexibility from staff (S) continued changes in shifts to facilitate deployment of staff to other hospitals (S) incomprehension of the use of external resources when some staff were working reduced hours (P) defending decisions made by hospital management (P) difficulty finding competent staff for the critical care area (P)	being positive with the staff despite personally feeling tense (S) movement of staff between hospitals and to remote working or reduced hours (S)
	Material resources	difficulty finding PPE (P)	difficulty finding PPE (P)
	The working environment	stressful and associated with war-like images (P) the surreal atmosphere substituting the care for patients that is usually given by family members (P)	
	The role held	managing a dual role—clinical and organisational (P) being in a new department performing activities that you haven’t done for many years (P)	
*Communication flow and adapting to the situation*	Information	information in excess, discordant and unclear (P) difficult to transmit clear timely information to staff which often caused confusion and uncertainty (P) trying to provide information and reassure staff regarding personal protective measures without creating panic (S) finding out information about the organisation from the media—that hospital departments were closing or opening (P)	information scarce and unclear, making it difficult to answer questions (P) information too much, discordant (P) reassuring staff about PPE use due to the lack of information (S) incoherent information caused by continually changing directives (P)
	Adaptation	having to rapidly adapt to new situations, directives and procedures to follow (P)	

P—Problems; PPE—Personal Protective Equipment; S—Strategies.

**Table 5:** Factors helpful in facing the pandemic.

Theme	Direct-Care Managers (*n* = 26) (Nurses/Doctors)	Non Direct-Care Managers (*n* = 9) (Administrative, Technical and Hospitality Service)
*Personal characteristics, knowledge and competency*	availability both personally and of their staff reciprocal trust staff professionalism colleagues knowledge made it possible to recognise and enhance each other’s knowledge and creativity adaptation, flexibility, resilience organisational skills, being concrete and rational, problem-solving novel situation and the need to deal with it knowledge of the context	the understanding of others is important staff professionalism
*Support from inside and outside of the organisation*	continuous exchange with the closest and trusted colleagues sharing and addressing issues together with other managers in weekly meetings team spirit support and backing from the line manager—being present and available concrete backing and the presence of senior management groups within the organisation support from non-healthcare staff from outside of the hospital support from family—a safe haven, returning to normality for a short while	collaboration between colleagues sharing through creation of a chat group support and backing from the line manager—being present and available presence of senior management groups within the organisation active presence of the Civil Protection within the hospital
*We’re all in the same boat*	positive working environment being united to face the many difficulties collaboration between nurses and doctors helps resolve problems ICU staff welcoming and supporting staff from other units during professionally difficult situations united towards the same goal support with and from colleagues—listening, sharing and support	positive working environment being united to face the many difficulties feeling part of the team gave motivation, drive, and energy to face any obstacle

ICU—Intensive Care Unit.

**Table 6:** Proposals to better manage a crisis situation.

Theme	Direct-Care Managers (*n* = 23) (Nurses/Doctors)	Non Direct-Care Managers (*n* = 8) (Administrative, Technical and Hospitality Service)
*Provide support*	encourage active communication between managers and teams where emotions and experiences can also be accommodated	psychological support for those who have experienced the virus at first hand
*Improving information and communication*	provide summaries for key information improve communication between hospitals and sectors so it is clear and accessible filter information so only information useful and pertinent arrives to the unit attention to content using clear and concise language coordinate between information sources to avoid redundant or conflicting information improve communication between healthcare professionals	use the hospital app to receive timely updates of documents
*Having an emergency plan in place*	devise an emergency plan which defines roles and spaces in the management of a crisis have a plan for intervention and for restarting that is in line with reality, defining the necessary resources have a Crisis Unit with a longer-term and strategic approach to ensure continuity in decisions taken and their application	devise an emergency plan with potential crisis scenarios which defines roles and spaces in the management of a crisis
*Awareness of resources*	have access to a pool of nurses the organisation should select or train leaders in Transformational Leadership encourage for team building between staff of different organizational levels improve the availability of PPE so they can be supplied rapidly promote team spirit	have more trained personnel that can substitute staff in order to face the situation in dire straits continue the visible presence of senior management in the hospitals have a pandemic warehouse that is always active consideration for the staff member and their family
*Attention to care*	showcase aspects of ‘what worked’ in the COVID-19 wards and sharing this practice as a learning exercise create a Pneumology Service that is transversal across hospitals	improve information for the population regarding the safety of non-COVID-19 hospitals to avoid missing care in patients with other conditions
*Avoiding bellic language*	avoid terms such as ‘fight the virus’, instead focus attention on what happens in hospital—‘caring for patients’	
*Value the role of professionals*	professionals demonstrated not only technical competency but also relational competence and commitment	
*Don’t create false expectations*	there are no absolute certainties in healthcare and it is important not to create false expectations	
*Respecting the rules*	ensure that rules are respected ensure the right people are involved in the decision making process and allowing contribution when rules are being developed	

PPE—Personal Protective Equipment.

## Data Availability

The data that support the findings of this study are available from the corresponding author upon reasonable request.

## Supplementary Materials

The following supporting information can be downloaded at: https://www.mdpi.com/article/10.3390/healthcare12040447/s1, File S1: Brief guideline for participants.
