# Supplementary material for: Managing the COVID-19 Pandemic: Experience of Managers in Healthcare: A Narrative Inquiry
Source: Healthcare (Basel). 2024 Feb 9;12(4):447. doi: 10.3390/healthcare12040447 (PMC10887853; doi:10.3390/healthcare12040447)
Supplement: Supplementary file 1 [file healthcare-12-00447-s001.zip › healthcare-2850210-supplementary.pdf]

Supplementary Material 1- Brief guidelines for participants

Dear colleague,

During the COVID-19 pandemic, all professional figures in the (*Hospital's Name*) were called upon to face a great challenge in which everyone made a substantial contribution in terms of the spirit of sacrifice, sense of mission, teamwork, skills, availability, and inventiveness. Particular commitment was required of those with management and leadership responsibilities at all levels.
With a view of enhancing the strategies adopted and analysing the problems encountered, **we kindly ask you to write a paper on your experience <u>by 31st August 2020</u>**.

We ask you to do so **in a free and spontaneous manner in the form of a story/narrative**, bringing to mind the aspects that you perceive as most important and significant for you, the people you care for, and the people with whom you collaborated and/or managed the activities under your responsibility.

In the course of your narrative, if it has not already spontaneously emerged, **we would ask you to also tell us about your experience with respect to the following guiding questions**:

- What were the main difficulties you found within your role, and what solutions did you find to address them?
- What were the main elements (personal resources, external help, other people, etc.) that helped you most in facing the difficulties you found?
- What suggestions for improvement do you have based on your experience?

Your anonymity will be guaranteed. Those who analyse your answers will have no way of knowing your identity.

Thank you for wanting to share and leave a trace of what you have experienced in this crisis situation! Your experience and feedback are very important to us, and we are sure that telling our story will benefit all of us.
